# Flavor Changes in Reduced-Salt Sour Meat During Synergistic Fermentation with Salt Substitutes and *Lactobacillus plantarum* SJ-4: Based on Microbial Community and Metabolomic Analyses

**DOI:** 10.3390/foods15111978

**Published:** 2026-06-02

**Authors:** Ning Liu, Ying Chen, Yuqian Guan, Lihua Shang, Chaoyue Yang, Cinan Li, Yuanyuan Liu, Qiujin Zhu, Ying Zhou

**Affiliations:** 1 Guizhou Key Laboratory of New Quality Processing and Storage of Ecological Specialty Food, School of Liquor and Food Engineering, Guizhou University, Guiyang 550025, China; 15120234350@163.com (N.L.); 15286181841@163.com (Y.C.); guanyq3514@163.com (Y.G.); 17785685052@163.com (L.S.); 18184191235@163.com (C.Y.); 19984462990@163.com (C.L.); yyliu3@gzu.edu.cn (Y.L.); ls.qjzhu@gzu.edu.cn (Q.Z.); 2 Key Laboratory Mountain Plateau Animals Genetics and Breeding, Ministry of Education, Guiyang 550025, China

**Keywords:** sour meat, reduced-salt, flavor, metabolomics, microorganism

## Abstract

This study investigated the effects of *Lactiplantibacillus plantarum* SJ-4 inoculation, partial replacement of sodium chloride (NaCl) with potassium chloride (KCl) and calcium ascorbate (CaA), and their combined treatment on the fermentation quality of fermented pork sour meat. Microbial community and metabolomic analyses were further integrated to explore the potential mechanisms related to flavor formation and quality changes. The combined treatment (LP + K-Ca group) improved the flavor profile, increased the contents of volatile compounds and free amino acids, reduced several undesirable flavor-related compounds, and contributed to better fermentation quality. Compared with the control group (C), total free amino acids and essential amino acids in the LP + K-Ca group increased by 4.95% and 22.71%, respectively, while the contents of undesirable compounds such as 2-nonenal, hypoxanthine, and ketones decreased by 73.65%, 73.40%, and 9.58%, respectively. Key flavor-related microorganisms included *Macrococcus*, *Staphylococcus*, and *Lactiplantibacillus*. These microorganisms are intricately linked to metabolic products such as amino acids, fatty acids, and organic acids, which may provide important precursors for volatile flavor formation. Overall, the combined treatment of 40% NaCl replacement with 24% KCl and 16% CaA, together with 2% *L. plantarum* SJ-4 inoculation, showed potential for improving flavor formation and fermentation quality in salt-reduced sour meat based on partial NaCl replacement.

## 1. Introduction

Sour meat is a distinctive traditional fermented meat product in Hunan, Guizhou and other regions of China, which is typically prepared from fresh pork, glutinous rice and salt via sealed fermentation. However, traditional sour meat processing generally requires relatively high salt addition to ensure flavor formation and fermentation stability. Previous studies have reported sodium chloride (NaCl) levels of approximately 4.0–6.0% (*w*/*w*) in traditional sour meat processing [1], which usually results in a salt level exceeding global dietary guidelines, thereby increasing health risks such as hypertension and kidney disease [2]. Furthermore, the long fermentation period easily leads to the growth of harmful microorganisms, posing potential food safety risks. Therefore, reducing the salt content of sour meat and enhancing the safety of the fermentation process have emerged as critical areas of research.

Salt reduction in meat product processing presents considerable challenges, because salt is not only critical for product flavor and texture, but also plays key roles in water-holding capacity, protein solubilization, and inhibition of harmful microorganisms. Therefore, reducing salt content may lower food safety, accelerate spoilage, and impair sensory quality and shelf life [3,4]. To tackle this challenge, researchers have been diligently investigating efficient salt reduction methods with the goal of decreasing sodium levels while preserving or enhancing product quality. Using non-sodium salts is a common strategy for reducing sodium levels in meat products. However, single salt substitutes, such as potassium chloride (KCl), may cause undesirable sensory defects, including bitterness and metallic taste [5,6]. Therefore, compound salt systems have attracted increasing attention as a way to reduce sodium content while maintaining product quality. For example, replacing 33% NaCl with a mixture of 22% KCl and 11% calcium ascorbate (CaA) significantly improved the flavor profile and sensory quality of bacon during storage [7], demonstrating the application potential of this strategy in sodium reduction for meat products.

During the fermentation of sour meat, microorganisms and endogenous enzymes collaboratively degrade proteins and lipids, generating flavor compounds and free amino acids (FAAs), thereby improving the nutritional value of the product [8,9]. Inoculation with *L. plantarum* starter culture contributes to reducing pH, inhibiting harmful microorganisms, promoting the release of FAAs and fatty acids, and enhancing flavor stability [10]. Previous studies have demonstrated that both starter culture inoculation and reduced-salt regulation can improve the quality of sour meat to a certain extent. However, most existing studies have focused on a single intervention factor, and there is a lack of systematic understanding regarding their synergistic effects within the same fermentation system [11,12]. Therefore, this study systematically compared the traditional fermentation process with single treatments, including inoculation with 2% *L. plantarum* SJ-4 alone and 40% NaCl replacement with the KCl-CaA blend alone, as well as their combined treatment, and investigated their effects on the fermentation characteristics and quality-related indices of sour meat.

In our previous study, the optimal fermentation conditions for sour meat were established by replacing 40% NaCl with 24% KCl and 16% CaA, combined with 2% inoculation of *L. plantarum* SJ-4. Under these conditions, the fermented sour meat exhibited the best overall sensory quality. Accordingly, the present study adopted a synergistic fermentation strategy involving 24% KCl, 16% CaA, and 2% inoculation of *L. plantarum* SJ-4, with the aim of alleviating flavor-related challenges commonly encountered in sour meat produced using a salt-reduction strategy. Volatile flavor compounds were characterized using GC-MS analysis. Furthermore, an integrated approach combining 16S rRNA gene sequencing and untargeted metabolomics was employed to investigate the dynamic succession of microbial communities and the associated metabolic variations. This multi-dimensional analytical framework was designed to simultaneously address critical limitations of traditional spontaneous sour meat fermentation, including poor process stability, long fermentation cycles, and the industrial demand for reduced-salt products. Collectively, this work provides a promising technical strategy for optimizing the fermentation process of reduced-salt sour meat.

## 2. Materials and Methods

### 2.1. Chemicals and Reagents

*L. plantarum* SJ-4 was used as the starter culture in this study. The strain was activated in MRS broth at 37 °C for 20 h and subcultured for three successive generations before use. The bacterial culture was then centrifuged at 6000× *g* for 10 min at 4 °C, and the supernatant was discarded. The cell pellets were washed twice with sterile 0.85% saline solution and resuspended in sterile saline to obtain the bacterial suspension for sour meat inoculation. The final inoculation level was adjusted to approximately 10^7^ CFU/g of meat sample.

NaCl, KCl, CaA, methanol, acetonitrile, formic acid, hydrochloric acid, sulfosalicylic acid, and other analytical reagents used in this study were of analytical or chromatographic grade, as required for each analysis. Deionized water was used throughout the experiment.

### 2.2. Sample Preparation

Fresh pork loin was purchased from Huimin Fresh Supermarket in Guiyang, Guizhou Province, China. Fresh pork loin (lean-to-fat ratio = 1:2) was sliced into 7 × 6 × 0.5 cm, mixed thoroughly, and evenly allocated into four groups. All groups were tumbled and marinated with respective salt treatments for 1 h, then further marinated at 4 °C for 1 h to ensure homogeneous salt distribution. The four treatment groups were designed as follows: group C (control, 4% NaCl); group LP (inoculation: 2.4% NaCl, inoculated with 2% *L. plantarum* SJ-4 after 1 h of marination); group K-Ca (substitution: 40% NaCl replaced by KCl-CaA blend, KCl:CaA = 3:2); group LP + K-Ca (combined treatment: same salt substitution as group K-Ca, followed by 2% *L. plantarum* SJ-4 inoculation after 1 h of marination). After marination, all samples were supplemented with 40% stir-fried golden glutinous rice, 2% Sichuan pepper, and 2% ginger (based on meat weight), then vacuum-packaged and fermented at 15 °C and 80% relative humidity. Samples were collected on days 0, 5, 15, and 25 for further analysis.

### 2.3. Determination of Physicochemical Indexes

#### 2.3.1. pH and Total Acidity

1 g of each sample was homogenized with 9 mL of deionized water (XHF-D, Ningbo Xinzhi Biotechnology Co., Ltd., Ningbo, China), and the pH value was recorded with a calibrated pH meter (PHS-3C, Lexi, Shanghai, China). Total acidity was assayed according to the method described by Zhang et al. [13].

#### 2.3.2. Nitrite Content

The sample preparation for nitrite determination followed the method described by Wu et al. [14]. The absorbance of the prepared samples was measured at 540 nm by spectrophotometer (F-2700, Hitachi, Hitachi, Japan).

### 2.4. Determination of E-Tongue and E-Nose

#### 2.4.1. E-Tongue

The determination method of electronic tongue was performed with reference to the protocol described by Lan et al. [15]. E-tongue analysis was performed using an electronic tongue instrument (Model: SA402B, INSENT Co., Ltd., Beijing, China) equipped with a professional probe.

#### 2.4.2. E-Nose

10 g of minced meat were weighed and placed in a 50 mL centrifuge tube, sealed, and incubated for 2 h [16]. Analysis was performed using a PEN3 electronic nose (AIRSENSE, Schwerin, Germany), which features 10 metal oxide sensors for detecting various odors (Table 1).

### 2.5. Determination of Volatile Organic Compounds (VOCs)

Volatile organic compounds in fermented sour meat were analyzed using headspace solid-phase microextraction combined with gas chromatography-mass spectrometry (HS-SPME-GC-MS). The determination of volatile flavor compounds was performed with minor modifications according to the method described by Qi et al. [17]. Briefly, 0.2 g of sample was weighed into a headspace vial (Agilent, Palo Alto, CA, USA), and extraction was performed with a 120 μm DVB/CWR/PDMS fiber for 15 min. The fiber was then thermally desorbed at 250 °C for 5 min. Helium was used as the carrier gas at a constant flow rate of 1.2 mL/min. The oven temperature program was set as follows: held at 40 °C for 3.5 min, raised to 100 °C at 10 °C/min, further increased to 180 °C at 7 °C/min, and finally ramped to 280 °C at 25 °C/min, where it was maintained for 5 min. The MS was operated with an ion source temperature of 230 °C and ionization energy of 70 eV. VOCs were identified using a self-built database based on published literature, selected authentic standards, retention indices, and confirmed retention times. Selected ion monitoring (SIM) was performed for accurate detection. For each compound, one quantitative ion and two to three qualitative ions were selected. A compound was identified when its retention time was consistent with the reference value and all selected ions were detected in the background-subtracted mass spectrum [18,19]. Quantification was performed using the quantitative ion, and the relative contents of VOCs were calculated by an isotope-internal-standard-based semi-quantitative method [20].

### 2.6. Determination of Amino Acids

Amino acids were analyzed by Zhang et al. [21]. A total of 1 g of sour meat was mixed with 50 mL of 0.01 M hydrochloric acid and homogenized, extracted for 30 min, and filtered. 2 mL of the filtrate were mixed with 2 mL of 8% sulfenic acid and allowed to stand for 15 min. The solution was then centrifuged at 4 °C and 10,000 rpm for 10 min, filtered through a 0.45 μm filter, and analyzed using an automatic amino acid analyzer (S-433D, Secam, Munich, Germany).

### 2.7. DNA Extraction and Sequencing

DNA was extracted from the samples using the cetyltrimethylammonium bromide (CTAB) method [10]. The variable regions of the 16S rRNA gene were amplified by PCR with barcoded primers targeting the corresponding sequences. The amplification conditions were as follows: initial denaturation at 98 °C for 1 min, followed by 30 cycles of denaturation at 98 °C for 10 s, annealing at 50 °C for 30 s, and extension at 72 °C for 30 s, with a final extension at 72 °C for 5 min. The PCR products were detected by electrophoresis on a 2% agarose gel. Sequencing libraries were constructed according to the protocol of the TruSeq^®^ DNA PCR-Free Sample Preparation Kit (Illumina, CA, USA) with index tags added. The libraries were sequenced on the Illumina NovaSeq platform to generate 250 bp paired-end reads.

### 2.8. Metabolite Analysis and Identification

Metabolomic analysis was conducted according to the protocol described by Choi et al. [22] and moderately modified to adapt to the present experimental system. A 20 mg homogenized sample was taken and added with 400 µL of methanol-water solution (7:3, *v*/*v*) containing isotope-labeled internal standards. Caffeine-13C3, testosterone-D3, L-tryptophan-d5, and 2-amino-3-(2-chlorophenyl) propanoic acid were used as internal standards in the positive ion mode, while hexanoic acid-D11, 2-amino-3-(2-chlorophenyl) propanoic acid, tetradecanoic-2,2-D2 acid, and L-tryptophan-d5 were used as internal standards in the negative ion mode. The mixture was vortexed at 1500 rpm for 5 min and kept in an ice bath for 15 min. After centrifugation at 12,000 rpm and 4 °C for 10 min, 300 µL of the supernatant was collected, incubated at −20 °C for 30 min, and centrifuged again under the same conditions for 3 min. Finally, 200 µL of the supernatant was harvested for LC-MS analysis.

Separation was performed on a Waters ACQUITY Premier HSS T3 column (Waters Corporation, MA, USA). Mobile phase A was 0.1% formic acid in water, and mobile phase B was 0.1% formic acid in acetonitrile. The same gradient elution program was used in both positive and negative ion modes: from 5% to 20% B over 1 min, then increased to 99% B over the next 2 min and held for 1.5 min, followed by a rapid return to 5% B in 0.1 min and equilibration for 1.4 min, with an injection volume of 4 µL. Mass spectrometry analysis was performed using an electrospray ionization source in both positive and negative ion modes. The ion spray voltage was set at 3.5 kV for positive ion mode and 3.2 kV for negative ion mode. Stepped collision energies of 30, 40, and 50 V were applied.

QC samples were prepared by pooling equal aliquots of all sample extracts to monitor the repeatability and stability of the LC-MS system. Raw MS data were converted to mzML format using ProteoWizard, followed by peak picking, peak alignment, and retention time correction using XCMS. For feature matching, the mass tolerance between MS1 and MS2 features was set at 25 ppm, and the retention time tolerance was set at 6 s. Peaks with missing values in more than 50% of samples within each group were removed, and missing values were filled using k-nearest neighbors (KNN) or one-fifth of the minimum value. Peak areas were corrected using the support vector regression (SVR) method. The metabolites were annotated by searching MetwareBio’s in-house database (https://www.metwarebio.com/, accessed on 26 September 2025), integrated public database, prediction database and metDNA. The mass tolerance was set at 25 ppm for precursor ion matching and 50 ppm for MS/MS spectral matching, and the retention time tolerance for database searching was set at 60 s. Most metabolites were putatively annotated at MSI level 2. Metabolites with an integrated annotation score >0.5 and a QC sample CV < 0.3 were retained for further analysis. Differential metabolites were screened using VIP > 1 and *p* < 0.05. Pathway analysis was conducted online via MetaboAnalyst 6.0 (https://www.metaboanalyst.ca/, accessed on 13 October 2025).

### 2.9. Statistical Analysis

Following analysis of variance (ANOVA), Duncan’s multiple range test was employed to identify statistically significant differences between groups, with a significance threshold set at *p* < 0.05, conducted with IBM SPSS Statistics 25 software (SPSS, Inc., Chicago, Illinois, USA). Figures were generated using Origin 2024 (OriginLab Corporation, USA). Data processing and statistical analysis of heatmaps for flavor compounds and metabolomics, as well as microbial diversity, community composition, and differential abundance analyses, were performed using R software (version 4.3.2). Principal component analysis (PCA) and orthogonal partial least squares discriminant analysis (OPLS-DA) were performed using SIMCA 14.1 (Umetrics, Umea, Sweden). The Redundancy analysis (RDA) was performed using Canoco 5 (Biometris Plant Research International, Wageningen University, Wageningen, The Netherlands). All experiments were performed in three parallel replicates, and the experimental results are expressed as mean ± standard deviation.

## 3. Results and Discussion

### 3.1. Changes in Physicochemical Properties of Sour Meat During Fermentation

#### 3.1.1. Dynamic Changes in pH and Total Acid Content

**Figure 1:**
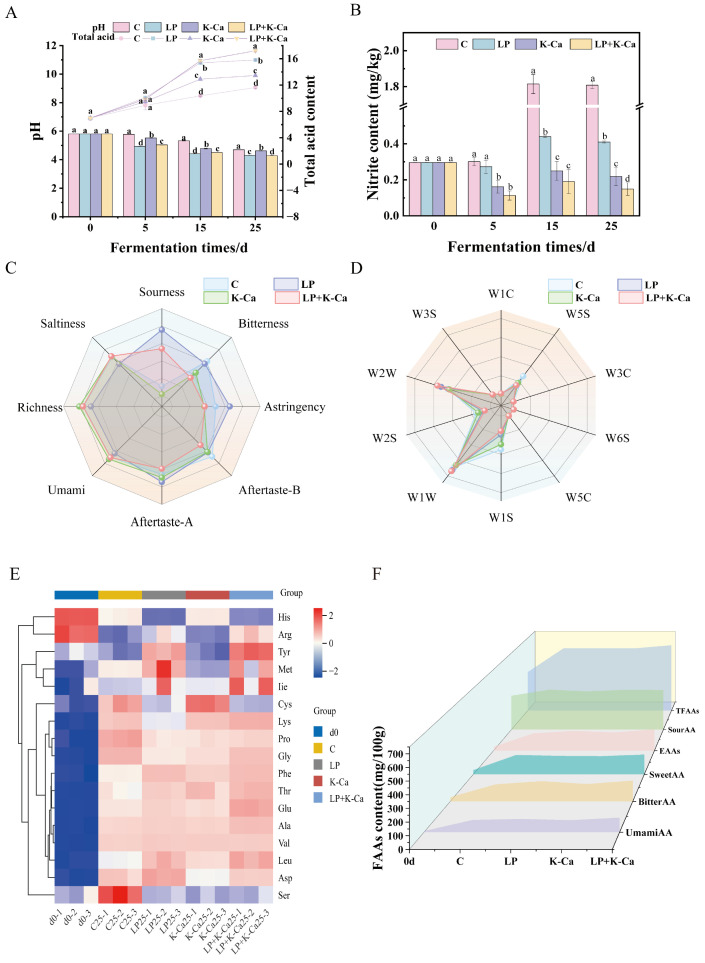
Changes in pH and total acidity (**A**), nitrite content (**B**), E-tongue (**C**), E-nose (**D**), heatmap of FAAs (**E**), and total taste-active amino acids (**F**) during the fermentation of sour meat. (UmamiAA is Glu and Asp; SourAA is His, Glu and Asp; BitterAA is Phe, Leu, Ile, Tyr, Pro, Arg, Met and Val; SweetAA is Pro, Met, Val, Lys, Ala, Gly, Ser and Thr; EAA is Thr, Val, Met, Iie, Leu, Phe, Lys; TFAAs is the total of all the above amino acids). 0d represents the sample on day 0. C (control group), LP (inoculation group), K-Ca (substitution group) and LP + K-Ca (combined treatment group). 0, 5, 15, and 25 represented fermentation time (day). Note: Different lowercase letters indicate significant differences between groups (*p* < 0.05).

During fermentation, pH values in all groups showed a consistent decline, with significant differences among treatments (*p* < 0.05) (Figure 1A). On the 25th day, the LP + K-Ca group reached a pH of 4.29, significantly lower than the C group (4.63) (*p* < 0.05). This rapid acidification is attributed to the dominance of inoculated *L. plantarum* SJ-4, which efficiently converts carbohydrates into organic acids like lactic acid [23]. The K-Ca group maintained a lower pH than the control throughout fermentation, suggesting that calcium ions may further reduce pH [24]. CaA likely enhances acidification by lowering redox potential, promoting lactic acid bacteria while inhibiting harmful microbes [25]. These results were consistent with the total acid changes, indicating that the combined treatment of 40% NaCl replacement with KCl-CaA and 2% *L. plantarum* SJ-4 inoculation promoted acidification during fermentation.

#### 3.1.2. Dynamic Change in Nitrite Content

The nitrite content exhibited an overall trend of initial decrease, followed by a slight increase, and a subsequent decrease (Figure 1B). This phenomenon can be attributed to the acidic environment provided during fermentation, which inhibits the growth of miscellaneous bacteria in meat and facilitates the degradation of nitrite [26]. During the fermentation process, the nitrite content in the LP + K-Ca group was significantly lower than that in the C group (*p* < 0.05), decreased by 91.75%. This may be due to two aspects. On the one hand, CaA is reductive and can directly reduce nitrite to nitric oxide, thus preventing its accumulation [27]. On the other hand, *L. plantarum* SJ-4 rapidly lowers the pH through lactic acid fermentation, which significantly inhibits the activity of nitrate reductase. In addition, lactic acid bacteria can produce nitrite reductase, enabling them to degrade nitrite [28]. These results indicate that KCl-CaA replacement combined with *L. plantarum* SJ-4 inoculation was more effective in reducing nitrite accumulation, which was consistent with the stronger acidification observed in the LP + K-Ca group.

### 3.2. Taste and Aroma Characteristics of Sour Meat at the End of Fermentation Based on E-Tongue and E-Nose Analyses

#### 3.2.1. Analysis of Taste Characteristics Based on E-Tongue

As shown in Figure 1C, both inoculation with *L. plantarum* SJ-4 alone and partial replacement of NaCl improved the flavor of sour meat, among which the LP + K-Ca group exhibited the optimal effect, characterized by increased umami and richness, as well as reduced bitterness, astringency and undesirable aftertaste. The elevated acidity was derived from the accumulation of lactic acid and acidic amino acids, while the decrease in pH might obscure the perception of saltiness, which explained the weakened saltiness in group LP [29]. The increase in umami and richness may be related to the accumulation of FAAs and flavor peptides during fermentation [30]. The diminished bitterness and astringency in the LP + K-Ca group could be attributed to the degradation of proteins and lipids, as well as the differences in microbial activity that affected the contents of bitter substances such as tryptophan and hypoxanthine [31].

#### 3.2.2. Analysis of Aroma Characteristics Based on E-Nose

**Figure 2:**
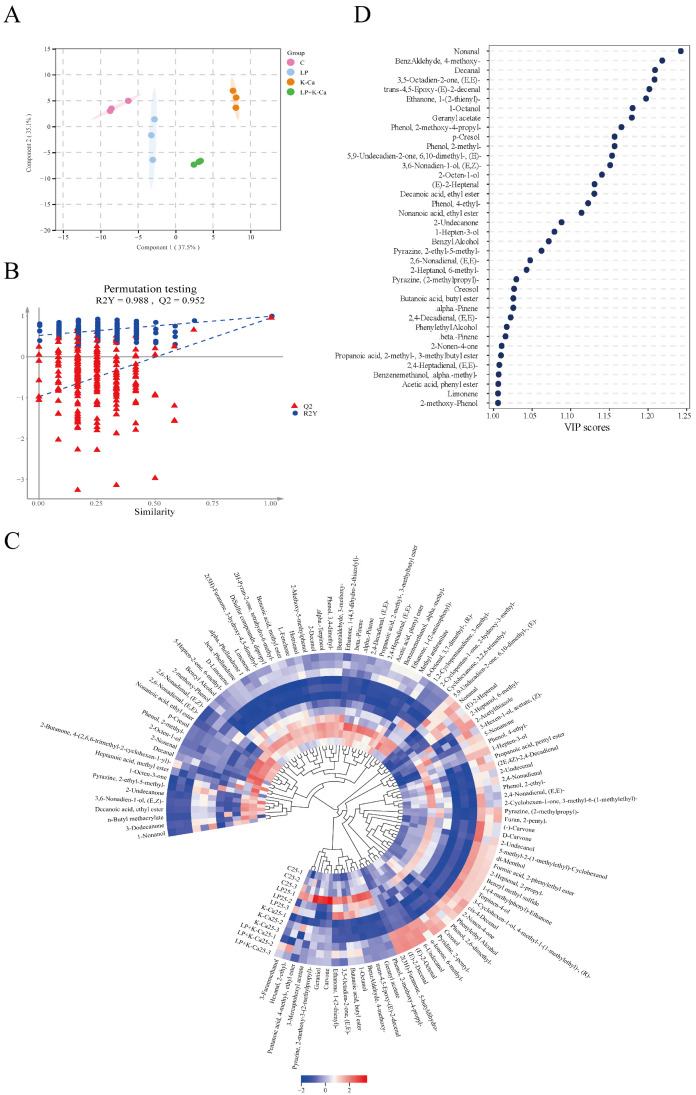
OPLS-DA analysis of VOCs in sour meat at the end of fermentation. Score diagram (**A**), permutations diagram (**B**), the dashed lines indicate the fitted regression lines of R^2^Y and Q^2^ obtained from permutation testing; heat map visualization and clustering results of the volatile compounds (**C**) and VIP scores (**D**).

The radar graphs of each sour meat sample at the end of fermentation showed significant differences in sensor responses (Figure 1D). Among them, the response intensities of sensors W1W, W2W, W1S and W5S were relatively high, indicating that aromatic compounds, alkanes, terpenoids, and sulfur-containing compounds were the major aroma contributors, which was consistent with the study by Cheng et al. [32]. The C group showed higher responses of W1S and W5S sensors, suggesting higher contents of nitrogen oxides and alkanes in the samples. Conversely, the LP + K-Ca group exhibited elevated responses of W1W and W2W, indicating increased contents of sulfides and aromatic compounds. These findings aligned with the outcomes of GC-MS analysis (Figure 2).

### 3.3. Composition and Content of FAAs in Sour Meat

During fermentation, FAAs derived from protein hydrolysis are key indicators for evaluating the quality of meat products [33]. Analysis of 17 FAAs (Table 2) showed that TFAA contents increased in all groups, with increases of 61.79%, 61.75%, 61.66%, and 69.80% in the C, LP, K-Ca, and LP + K-Ca groups, respectively. Notably, the LP + K-Ca group showed a significantly higher TFAA content than the other groups (*p* < 0.05). According to taste characteristics, FAAs can be classified into umami amino acids (Umami AA), sour amino acids (Sour AA), sweet amino acids (Sweet AA), bitter amino acids (Bitter AA), and essential amino acids (EAAs). Their composition and contents collectively determine the final flavor profile of the product [34]. As shown in Figure 1E,F, compared with the C group, the LP + K-Ca group had significantly higher contents of Asp and Glu, as well as Ala and Lys (*p* < 0.05), indicating greater accumulation of taste-active amino acids. In addition, EAAs were significantly increased, suggesting improved nutritional value. It has been reported that KCl can enhance the activities of cathepsins B and B + L and prolong protein degradation [35], while *L. plantarum* SJ-4 promotes protein decomposition and amino acid release [36]. Therefore, 40% NaCl replacement with KCl-CaA combined with 2% *L. plantarum* SJ-4 inoculation promoted FAA accumulation and provided more taste-active compounds for sour meat flavor development.

### 3.4. VOC Profiling of Sour Meat at the End of Fermentation by GC-MS

#### 3.4.1. Identification and Quantification of VOCs

The OPLS-DA results showed significant differences in VOCs among the four groups of sour meat (Figure 2A). The results of the permutation test confirmed the validity of the analytical model (Figure 2B). A total of 105 VOCs were identified by GC-MS, including 21 alcohols, 24 ketones, 21 aldehydes, 15 esters, 10 phenols, and 14 other compounds (including heterocyclic compounds, ethers and terpenes) (Table 3). The LP + K-Ca group demonstrated a greater overall concentration of VOCs compared to the remaining experimental groups, and the combined treatment increased the contents of alcohols, aldehydes and heterocyclic compounds.

The formation of alcohols in fermented meat is closely associated with microbial carbohydrate fermentation and aldehyde reduction. The alcohol content in the LP + K-Ca group was significantly higher than the K-Ca group (*p* < 0.05), likely due to the partial substitution of NaCl with KCl and CaA, reducing the antibacterial effects of NaCl, and the enhanced microbial activity from *L. plantarum* SJ-4 [3]. Notably, the LP + K-Ca group exhibited a significantly higher content of phenylethyl alcohol, an aromatic compound, compared to the other groups (*p* < 0.05). This substance is generated through microbial-mediated amino acid metabolism and imparts a sweet rose aroma [11], which is consistent with the electronic nose results. Furthermore, monoterpene alcohols such as terpinen-4-ol and (R)-4-methyl-1-(1-methylethyl)-3-cyclohexen-1-ol of LP + K-Ca group were also present in higher abundances. These substances are typically derived from spices, imparting peppery, woody and floral notes [37].

Aldehydes are generally regarded as important aroma-active compounds in fermented meat products due to their relatively low odor thresholds. Their formation is closely related to lipid oxidation and degradation, amino acid-derived Strecker degradation, Maillard reactions, and microbial metabolism during fermentation [38]. In this study, 21 aldehydes were detected, and the LP + K-Ca group showed the highest total aldehyde content, which was significantly higher than that of the K-Ca group (*p* < 0.05). This change may be associated with the combined regulation of salt-ion environment, microbial succession, and *L. plantarum* SJ-4-mediated metabolism, which together affect lipid degradation and aldehyde formation during fermentation [39,40,41,42]. As illustrated in the heatmap (Figure 2C), the concentrations of unsaturated aldehydes, including (E)-2-Heptenal and (E)-2-Octenal, were markedly elevated in the LP + K-Ca group relative to the C group (*p* < 0.05). These compounds possess fatty and fruity characteristics, exerting a significant effect on flavor enhancement [43]. These lipid oxidation-derived aldehydes interact with Maillard reaction intermediates, facilitating the formation of heterocyclic sulfur-containing compounds [44], which is consistent with the electronic nose results (Figure 1D). Notably, the content of 2-nonenal, a typical off-flavor compound derived from unsaturated fatty acid oxidation, was significantly decreased in the LP + K-Ca group (*p* < 0.05), decreased by 73.66%. This change helped reduce the production of lipid oxidation-derived off-flavors, exerting a positive effect on maintaining the flavor and overall quality of sour meat [45].

Although there was no statistically significant variation in total ester content between the LP + K-Ca group and the C group, both groups exhibited significantly higher ester levels than the LP and K-Ca groups (*p* < 0.05). This indicated that the synergistic effect of *L. plantarum* SJ-4 and salt substitutes promoted the synthesis of esters. This might be attributed to the reduced inhibition of bacterial growth caused by low sodium content [3], which combined with the targeted regulation of the starter culture, enhanced esterase activity in sour meat. Notably, the contents of butanoic acid, butyl ester and formic acid, 2-phenylethyl ester in the LP + K-Ca group were significantly higher than those in the C group (*p* < 0.05), increased by 36.84% and 23.23%, respectively, contributing fruity, rosy and fresh aromas.

Methyl ketones such as 2-undecanone, ethanone and 1-octen-3-one are derived from fatty acid β-oxidation and contribute fruity, creamy and mushroom aromas to fermented products [46]. However, excessive ketone content may lead to flavor deterioration [47]. Compared with the C group, the total ketone level in the LP + K-Ca group was significantly decreased by 9.58% (*p* < 0.05). This may be associated with the antioxidant effect of CaA and the acidic environment created by *L. plantarum* SJ-4, which could limit excessive lipid-derived oxidation reactions. Such regulation of ketone accumulation helps maintain flavor balance and prevent the formation of undesirable odors.

Phenols, terpenoids, heterocyclic compounds, and sulfur-containing compounds also contributed to the aroma profile of sour meat. A total of 10 phenolic compounds were identified, including 2-methoxy-4-propyl-phenol, 4-ethyl-phenol, and 3,4-dimethyl-phenol, which may provide pungent, slightly sour, and roasted notes [48]. Terpenoids, such as β-pinene, α-pinene, and limonene, contributed sweet and pleasant aromas, while alkylpyrazines, including 2-methoxy-3-(2-methylpropyl)-pyrazine and 2-methylpropyl-pyrazine, were associated with peppery and roasted aromas, probably partly derived from spices such as Sichuan pepper. Compared with the C group, the total content of heterocyclic compounds in the LP + K-Ca group increased by 15.63% (*p* < 0.05), indicating that the combined treatment promoted the formation of characteristic aroma-active compounds. In addition, sulfur-containing compounds in the LP + K-Ca group increased by 18.23% compared with the C group (*p* < 0.05), which was consistent with the E-nose results. Benzyl methyl sulfide, dipropyl disulfide, and 2-acetylthiazole are sulfur-containing flavor compounds with low odor thresholds and strong aroma activity, contributing meaty, roasted, sulfurous, and fermented notes to the characteristic flavor of meat products [49]. These differences may be attributed to the changes in protein structure and physicochemical properties caused by the partial replacement of NaCl with KCl and CaA followed by the inoculation of *L. plantarum* SJ-4 [7].

#### 3.4.2. Screening of Key VOCs

OPLS-DA analysis identified variables with VIP > 1 and *p* < 0.05 as significant for distinguishing sample differences. As shown in Figure 2D, 38 compounds exhibited VIP > 1, including 8 alcohols, 5 ketones, 8 aldehydes, 6 esters, 6 phenols and 5 other compounds (comprising heterocyclic compounds and terpenoids). These compounds are the main compounds contributing to the flavor differences in sour meat and can serve as key indicators for flavor regulation and quality optimization.

### 3.5. Dynamic Changes in the Microbial Community of Sour Meat During Fermentation

#### 3.5.1. Differences in Microbial Community Structure Based on Alpha and Beta Diversity

**Figure 3:**
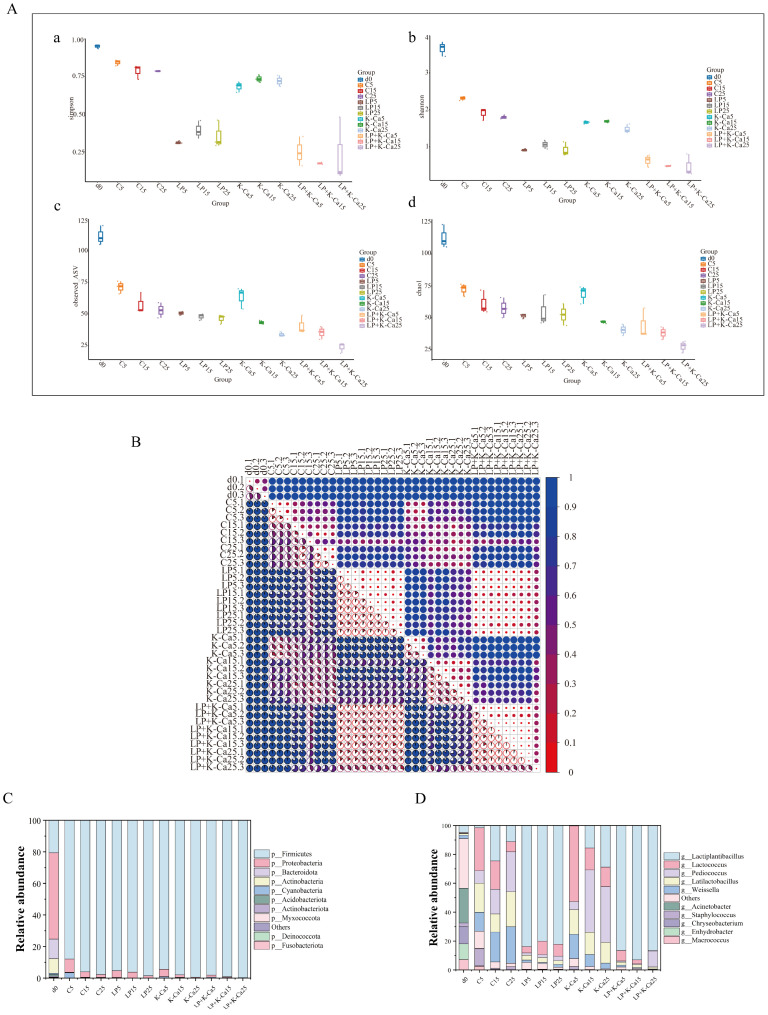

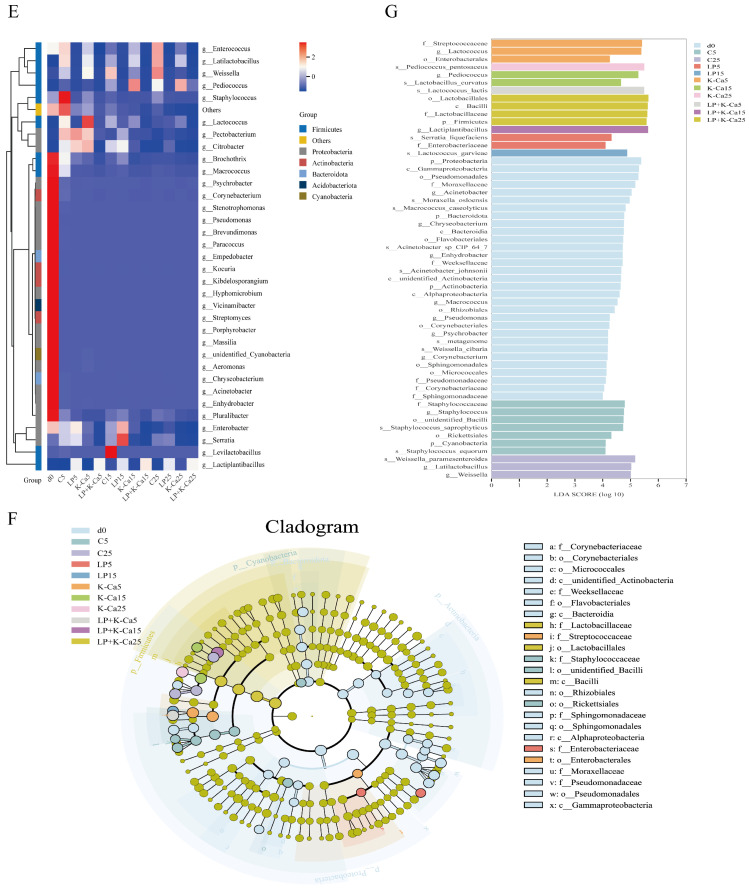
Box plot of index difference between groups of observed Simpson (**a**), Shannon (**b**), ASV (**c**), Chao1 (**d**) in sour meat (**A**). Heatmap of beta diversity indices (**B**), the smaller and redder the circle, the lower the beta diversity value and the smaller the diversity difference among samples. The color and size of the lower triangles follow the same rules as those of the upper triangles. Changes in the relative abundance of the microbial community of sour meat at the phylum level (**C**) and the genus level (**D**). Top 35 genera annotated according to the species and abundant information (**E**). The evolutionary branch map (**F**) and LEfSe analysis (**G**).

Microbial diversity was evaluated using the Shannon and Simpson indices, while microbial richness was quantified through Amplicon Sequence Variant (ASV) counts and the Chao1 indices. As shown in Figure 3A, samples at day 0 exhibited relatively high microbial diversity and richness, likely originating from the raw meat and processing environment. During fermentation, microbial diversity decreased significantly, especially in groups LP and LP + K-Ca. This reduction may be attributed to nutrient depletion, the inability of exogenous microorganisms to adapt to the increasingly acidic environment, and the inhibition of other microbes by lactic acid bacteria as the dominant flora [50]. The simplified microbial community structure and consistent metabolic pathways contributed to the controlled safety of sour meat.

Microbial community analysis (Figure 3B) revealed that the microbial diversity in the C, LP, K-Ca and LP + K-Ca groups differed significantly from that observed on day 0. The LP + K-Ca and LP groups showed similar community structures, as did the C and K-Ca groups. However, marked differences existed between these two clusters, indicating that inoculation with *L. plantarum* SJ-4 exerted a greater impact on microbial composition than salt substitution. *L. plantarum* SJ-4 enhanced the dominance of lactic acid bacteria, inhibited harmful bacterial flora by accelerating acidification and secreting bacteriocins, improved the bacterial quality of sour meat, and promoted flavor development [10]. In contrast, the effects of KCl and CaA were relatively mild, mainly by increasing the abundance of *Lactobacillus*, *Lactococcus*, and *Pediococcus* to lower pH and facilitate fermentation.

#### 3.5.2. Microbial Community Composition at the Phylum and Genus Levels

At the phylum level (Figure 3C), the initial microbiota was dominated by *Proteobacteria* (54.7%), *Firmicutes* (20.6%), *Bacteroidetes* (12.4%) and *Actinobacteriota* (9.1%). As fermentation progressed, the relative abundance of *Proteobacteria* decreased sharply, reaching 1.8%, 1.3%, 0.4% and 0.1% in the C, LP, K-Ca, and LP + K-Ca groups on day 25, respectively. In contrast, *Firmicutes* increased significantly after 5 days of fermentation and accounted for more than 85% in all groups throughout fermentation, which was consistent with the findings of Lv et al. [51]. The anaerobic and low-pH environment during sour meat fermentation favored the growth of acid-tolerant facultative anaerobic lactic acid bacteria belonging to *Firmicutes* [52]. The accumulation of lactic acid further strengthened their dominant position and maintained the stability of the microbial ecosystem.

As shown in Figure 3D, the initial samples exhibited high microbial diversity at the genus level and were dominated by *Acinetobacter* (24.0%), *Micrococcus* (7.2%), and other genera (34.5%). This diverse initial microbiota may have originated from raw materials and the processing environment. As fermentation proceeded, the community structure became more concentrated, with the relative abundances of lactic acid bacteria, including *Lactobacillus*, *Lactococcus*, *Pediococcus*, and *Weissella*, gradually increasing. At the end of fermentation, the dominant genera differed among treatment groups. The C group was mainly composed of *Weissella* (25.9%), *Latilactobacillus* (24.8%), and *Pediococcus* (28.4%). The LP group was dominated by *Lactiplantibacillus* (82.2%) and *Lactococcus* (8.3%). In the K-Ca group, *Lactobacillus* (43.2%), *Pediococcus* (38.6%), and *Lactococcus* (13.5%) were the main genera, whereas the LP + K-Ca group was dominated by *Lactiplantibacillus* (86.6%) and *Pediococcus* (11.1%). These results were consistent with the findings reported by Yang et al. [53], indicating that lactic acid bacteria gradually became the dominant microorganisms during sour meat fermentation. The differences in dominant genera may be caused by salt composition and *L. plantarum* SJ-4 inoculation. Partial replacement of NaCl with KCl and CaA can modify bacterial community structure in meat products and promote the growth of lactic acid bacteria [54,55]. Therefore, the relative abundances of *Pediococcus*, *Lactococcus* and *Lactobacillus* increased in the K-Ca group. Compared with salt substitution alone, *L. plantarum* SJ-4 inoculation more strongly modulated microbial succession by promoting acidification and enhancing the competitive dominance of *Lactiplantibacillus*, which may improve microbial stability through the inhibition of undesirable microorganisms [56]. In addition, lactic acid bacteria such as *Lactococcus*, *Pediococcus*, and *Lactiplantibacillus* are closely involved in carbohydrate, protein, and lipid metabolism, contributing to the formation of organic acids, peptides, FAAs, fatty acids, and volatile compounds during fermentation [42,57,58].

#### 3.5.3. Microbial Community Composition and Biomarker Analysis

To further illustrate the microbial changes during sour meat fermentation, a cluster heatmap of the top 35 genera based on relative abundance at the genus level was constructed (Figure 3E). On day 0, the samples showed relatively high microbial abundance and contained 25 detectable genera. With fermentation progression, the microbial community became more simplified and gradually shifted toward a lactic acid bacteria-dominated structure. During fermentation, the C group was mainly associated with *Staphylococcus*, *Lactobacillus*, and *Weissella*, whereas the LP + K-Ca group maintained *Lactiplantibacillus* as the dominant genus from day 5 onward. The K-Ca group showed relatively high abundances of *Lactococcus* and *Pediococcus*, this may be related to the altered salt-ion environment caused by KCl-CaA replacement, which favored the growth of acid-tolerant lactic acid bacteria [11].

LEfSe analysis further identified microbial biomarkers at multiple taxonomic levels. The evolutionary cladogram (Figure 3F) showed that the day 0 samples were characterized by *Bacteroidetes*, *Proteobacteria*, and *Actinobacteriota*, whereas *Firmicutes* became the key biomarker at the end of fermentation. The LDA results (Figure 3G) showed that *Acinetobacter*, *Chryseobacterium*, *Enhydrobacter*, and *Macrococcus* were the major biomarkers in the initial samples. At the end of fermentation, different characteristic biomarkers were observed among treatment groups. The C group was characterized by *Weissella* and *Latilactobacillus*. As a heterofermentative lactic acid bacterium, *Weissella* can metabolize carbohydrates via the phosphoketolase pathway to produce lactic acid, ethanol, and CO_2_, which may contribute to acidification and aroma complexity in fermented meat products [59]. In group K-Ca, *Pediococcus* served as the biomarker. The addition of KCl and CaA accelerated the pH decline, favoring the growth of more acid-tolerant *Pediococcus* [11]. Certain strains of *Pediococcus* exhibit strong proteolytic activity, promoting the generation of soluble peptides and FAAs [58]. In the LP + K-Ca group, *Lactiplantibacillus* was identified as the dominant biomarker, consistent with the beta-diversity result that *L. plantarum* SJ-4 inoculation exerted a stronger influence on microbial community structure than salt substitution. This suggests that starter inoculation was the main factor driving the dominance of *Lactiplantibacillus* under the combined treatment. *Lactiplantibacillus* can adapt to acidic fermentation environments and participate in protein, lipid, and carbohydrate metabolism, thereby facilitating the formation of flavor precursors and volatile compounds [55]. Overall, the results suggested that KCl-CaA substitution mainly shaped the community by altering salt-related environmental pressure, whereas *L. plantarum* SJ-4 inoculation promoted a more directed lactic acid bacteria-dominated fermentation.

### 3.6. Evaluation of Differential Metabolites and Metabolic Pathways in Sour Meat at the End of Fermentation

**Figure 4:**
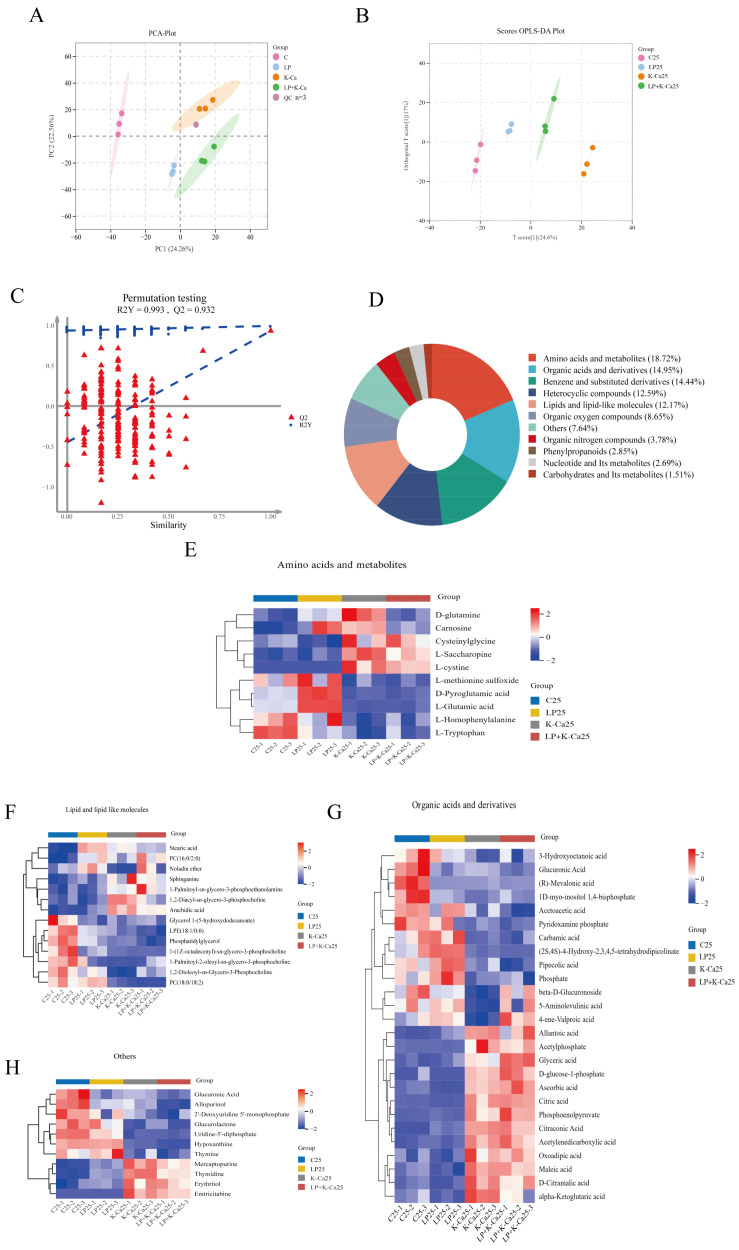

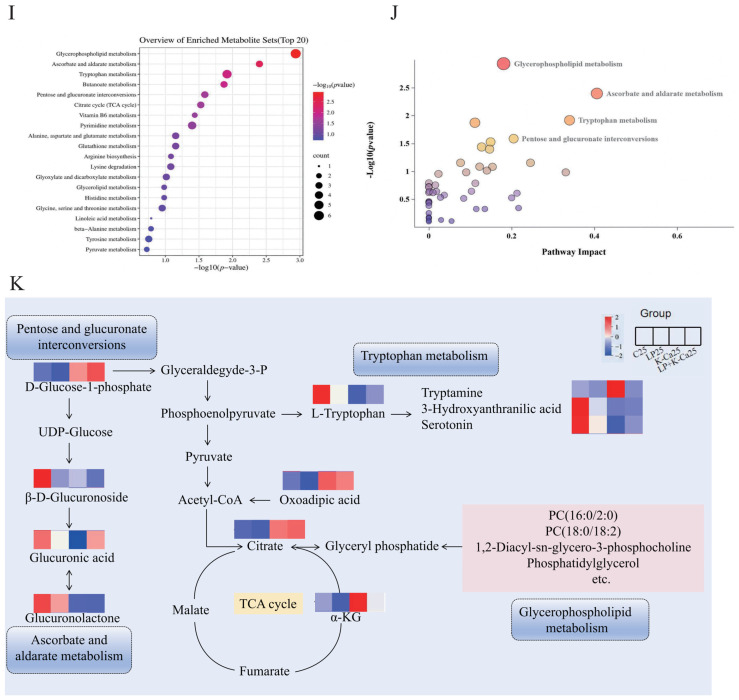
PCA based on metabolites of sour meat (**A**), OPLS-DA analysis (**B**) and permutations diagram (**C**), dashed lines indicate the fitted regression lines of R^2^Y and Q^2^ obtained from permutation testing. Metabolite classes and relative abundance percentages of sour meat (**D**). Clustering heat map of differential metabolites of sour meat (**E**–**H**). KEGG pathway analysis of sour meat in different groups (**I**–**K**). (**I**) Enrichment pathways for differential metabolites (Top 20). (**J**) Key metabolic pathways implicated in the metabolic changes, bubble size represents pathway impact, and bubble color indicates enrichment significance, with redder colors corresponding to lower *p*-values and higher significance. (**K**) Putative metabolic mechanism underlying the regulation of flavor formation in sour meat by the combined treatment of KCl-CaA, partial NaCl replacement and *L. plantarum* SJ-4 inoculation.

To investigate the effects of salt substitutes and *L. plantarum* SJ-4 on metabolic pathways during sour meat fermentation, non-targeted metabolomics techniques were performed on samples at the fermentation endpoint. To evaluate the stability and repeatability of the metabolomic data, quality control (QC) samples were introduced in the PCA. Meanwhile, the three QC samples were highly clustered and located near the center of the sample distribution in the PCA score plot (Figure 4A), indicating good repeatability and stability of the detection system. Furthermore, PCA results also showed distinct clustering with almost no overlap among the treatment groups, indicating that the metabolite profiles of sour meat had significant changes after treatment with salt substitutes and starter culture inoculation. OPLS-DA analysis (Figure 4B) and validation plots (Figure 4C) further confirmed the intergroup differences. A total of 1191 differential metabolites were identified across the four groups, which were classified into 11 main categories (Figure 4D). These included amino acids and metabolites (18.72%), organic acids and derivatives (14.95%), benzene and substituted derivatives (14.44%), heterocyclic compounds (12.59%), lipids and lipid-like molecules (12.17%), organic oxygen compounds (8.65%), organic nitrogen compounds (3.78%), phenylpropanoids (2.85%), nucleotides and their metabolites (2.69%), carbohydrates and their metabolites (1.51%) and others (7.64%).

#### 3.6.1. Composition and Classification of Differential Metabolites

Differential metabolites among groups were screened using the OPLS-DA model, with a VIP value > 1 and *p* < 0.05 as the threshold for statistical significance. Cluster analysis was performed on the screened significantly differential metabolites, and the results are displayed in the heatmaps (Figure 4E–H).

##### Analysis of Amino Acids and Metabolites

As shown in Figure 4E, the concentration of the Bitter AA L-tryptophan in the C group at the end of fermentation was significantly higher than in the other treatment groups (*p* < 0.05), increased by 14.79% compared with group LP + K-Ca, which was consistent with the highest bitterness score observed for this group in the E-tongue analysis (Figure 1C). In contrast, the Umami AA L-glutamic acid was significantly elevated in group LP (*p* < 0.05), contributing to enhanced umami flavor [60]. In groups K-Ca and LP + K-Ca, the contents of cysteinylglycine, L-saccharopine, and L-cystine were higher than in groups C and LP. These compounds are precursors of cysteine and can be converted into sulfur-containing compounds, which are critical to product flavor [61]. In addition, amino acid metabolism can also participate in the formation of aldehydes, alcohols, and esters through transamination, decarboxylation, and Strecker degradation, thereby increasing the contents of aromatic alcohols and esters, such as phenylethyl alcohol and formic acid, 2-phenylethyl ester, in the LP + K-Ca group [42]. These results suggested that amino acid metabolism contributed to both taste formation and VOC generation under the combined treatment.

##### Analysis of Lipids and Lipid-like Molecules

Lipids in sour meat were mainly composed of glycerophospholipids (Figure 4F). The content of saturated fatty acids (SFAs) in the LP + K-Ca group was significantly higher than that in the C group (*p* < 0.05), while the content of unsaturated fatty acids (UFAs) was significantly lower (*p* < 0.05). These differences might be attributed to the fact that KCl reduced the antibacterial activity of NaCl, enhanced microbial and enzymatic activities, and thus preferentially degraded UFAs with poor stability to generate flavor substances [3]. In addition, the distinct microbial metabolism induced by inoculation with *L. plantarum* SJ-4 might further affect fatty acid composition [42]. Lipid and glycerophospholipid-related metabolites are important sources of fatty acids and lipid-derived volatile compounds, and their changes may be associated with the formation of aldehydes, alcohols, ketones, and heterocyclic compounds in fermented meat products [38]. Therefore, the altered lipid metabolite profile in the LP + K-Ca group was consistent with the changed abundance of lipid-derived aldehydes observed in the VOC analysis, particularly (E)-2-heptenal and (E)-2-octenal, which were markedly elevated in this group. Meanwhile, the lower level of unstable unsaturated lipid-related metabolites in the LP + K-Ca group was in line with the reduced contents of lipid oxidation-related undesirable volatiles, such as 2-nonenal and total ketones, since UFAs are important substrates for the formation of secondary lipid oxidation products in meat systems [62]. Since SFAs are less susceptible to lipid peroxidation than UFAs, this shift may contribute to improved oxidative stability [63].

##### Analysis of Organic Acids and Derivatives

Organic acids contribute to regulating the microecological environment, imparting a unique flavor to sour meat, and improving its nutritional value [64]. Fermentation led to an increase in organic acid content, which was significantly higher in the LP + K-Ca group than in the C and LP groups (*p* < 0.05) (Figure 4G). Compared with group C, group LP + K-Ca increased by 20.93%. This increase contributed to a lower pH and accelerated fermentation. Additionally, these organic acids were further converted into other flavor compounds through microbial metabolism [65]. This may be attributed to partial replacement of NaCl with KCl and CaA, which reduced the Na^+^ concentration, and K^+^ as a key metabolic ion, enhanced the glycolysis and acid-producing capacity of lactic acid bacteria [66]. Specifically, the concentrations of ascorbic acid and glyceric acid in the LP + K-Ca group were significantly higher than those in the other groups (*p* < 0.05). Ascorbic acid prevents lipid oxidation and improves product color [67]. Glyceric acid and its phosphorylated derivatives act as core intermediates in the glycolysis pathway, which facilitates the generation of flavor precursors [68]. Furthermore, the contents of citric acid, α-Ketoglutaric acid, phosphoenolpyruvate, and D-glucose-1-phosphate in groups K-Ca and LP + K-Ca were higher than those in groups C and LP. These metabolites are closely associated with glycolysis and the tricarboxylic acid cycle (TCA cycle), and their accumulation may indicate enhanced central carbon metabolism and microbial activity during fermentation [69], which may support the formation of flavor-active compounds such as phenylethyl alcohol, formic acid, 2-phenylethyl ester, and butanoic acid, butyl ester.

##### Analysis of Other Metabolites

Nucleotides and their derivatives are key taste components (Figure 4H). The content of hypoxanthine, a bitter compound, was significantly higher in the C and LP groups than in the other two groups (*p* < 0.05), which was consistent with the stronger bitterness perception observed in these two groups in E-tongue analysis (Figure 1C) [70]. In contrast, hypoxanthine content was significantly decreased by 73.40% in the LP + K-Ca group, which likely improved flavor quality by reducing undesirable bitterness.

#### 3.6.2. KEGG Pathway Enrichment Analysis and Identification of Key Metabolic Pathways

To further understand the potential metabolic pathways of sour meat fermentation, differential metabolites were mapped to the KEGG pathway database, presenting the top 20 pathways ranked by *p*-value (Figure 4I). Generally, pathways with a *p*-value < 0.05 and an impact value > 0.1 are considered significant. Table 4 lists the top ten metabolic pathways with the highest impact. As shown in Figure 4J, four metabolic pathways related to the flavor and quality of sour meat were identified, including ascorbate and aldarate metabolism, tryptophan metabolism, pentose and glucuronate interconversions, and glycerophospholipid metabolism. These pathway results were consistent with the metabolite profile changes described previously. Ascorbate and aldarate metabolism highlighted the role of ascorbic acid in the LP + K-Ca group, while tryptophan metabolism reflected the variation in L-tryptophan and tryptamine. Pentose and glucuronate interconversions pointed to the involvement of carbohydrate-related metabolites, such as D-glucose-1-phosphate, in central carbon metabolism. Glycerophospholipid metabolism further indicated lipid remodeling, as reflected by changes in PC and LPE species.

As shown in Figure 4K, the D-glucose-1-phosphate content in the LP + K-Ca group was significantly higher than that in the C group (*p* < 0.05). As a key intermediate in glucose metabolism, D-glucose-1-phosphate can enter glycolysis after conversion to glucose-6-phosphate, thereby supporting pyruvate formation and subsequent flavor-related reactions [71]. In addition, α-ketoglutarate links the TCA cycle with amino acid metabolism and can be converted into the umami amino acid Glu through transamination [72]. This leads to a significant increase in Glu, consistent with the results of FAAs (Figure 1E).

Tryptamine, a type of biogenic amine, is produced from tryptophan via decarboxylation, and its excessive accumulation is generally regarded as an indicator of quality deterioration and increased putrid off-flavors [73]. Compared with the C and LP groups, the contents of tryptamine in the LP + K-Ca group were significantly decreased (*p* < 0.05), indicating that the combined treatment effectively inhibited the tryptophan decarboxylation pathway and reduced the formation of undesirable flavor compounds. Furthermore, the combined treatment also markedly influenced glycerophospholipid metabolism, which represents another key pathway involved in flavor regulation. Compared with the C group, the LP + K-Ca group showed significant accumulation of PC (16:0/2:0) and lysophosphatidylethanolamine LPE (16:0), but markedly decreased levels of PC (18:0/18:2) and phosphatidylglycerol (*p* < 0.05). As a flavor precursor generated by the preferential hydrolysis of sn-2 UFAs via lipase A2, the accumulation of PC (16:0/2:0) reflects the targeted regulation of lipid metabolism [74]. Meanwhile, the reduction in polyunsaturated fatty acid PC (18:0/18:2) lowers the risk of lipid peroxidation [75], thereby contributing to optimized flavor profiles and enhanced oxidative stability. Furthermore, glycerol-3-phosphate, a phospholipid metabolic intermediate, can enter glycolysis via glycerokinase, strengthening the synergy between glucose and lipid metabolism [76]. Overall, these pathway changes suggested that amino acid, organic acid, and lipid metabolism were the main metabolic processes associated with flavor differences among treatments.

### 3.7. Correlation Analysis of Key VOCs, Differential Metabolites, and Dominant Microorganisms in Sour Meat

**Figure 5:**
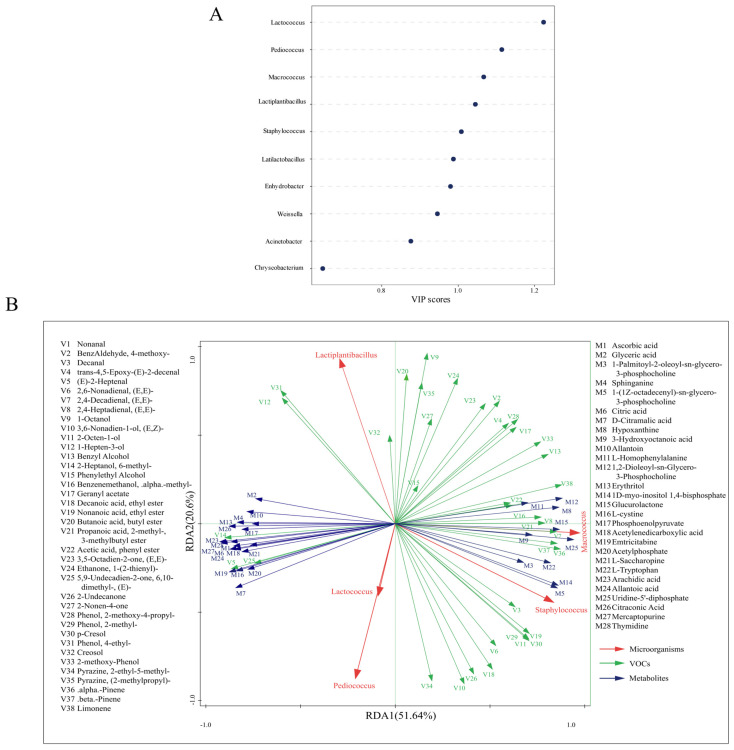

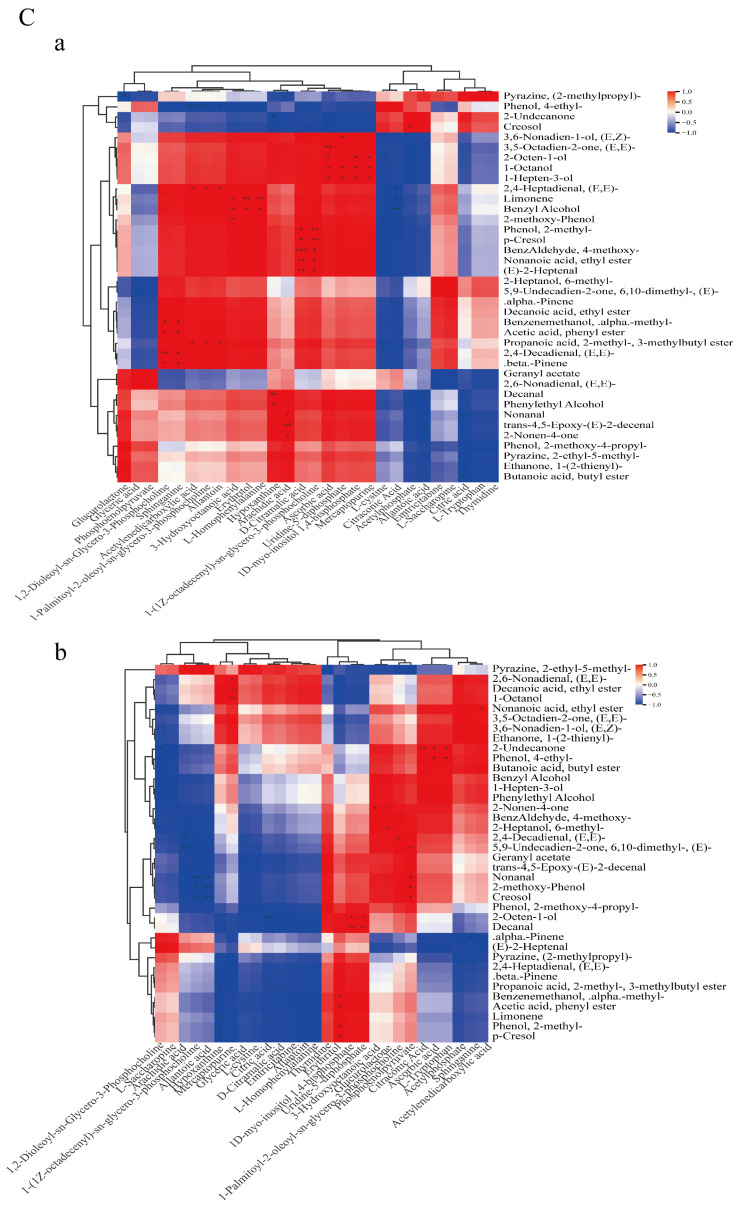

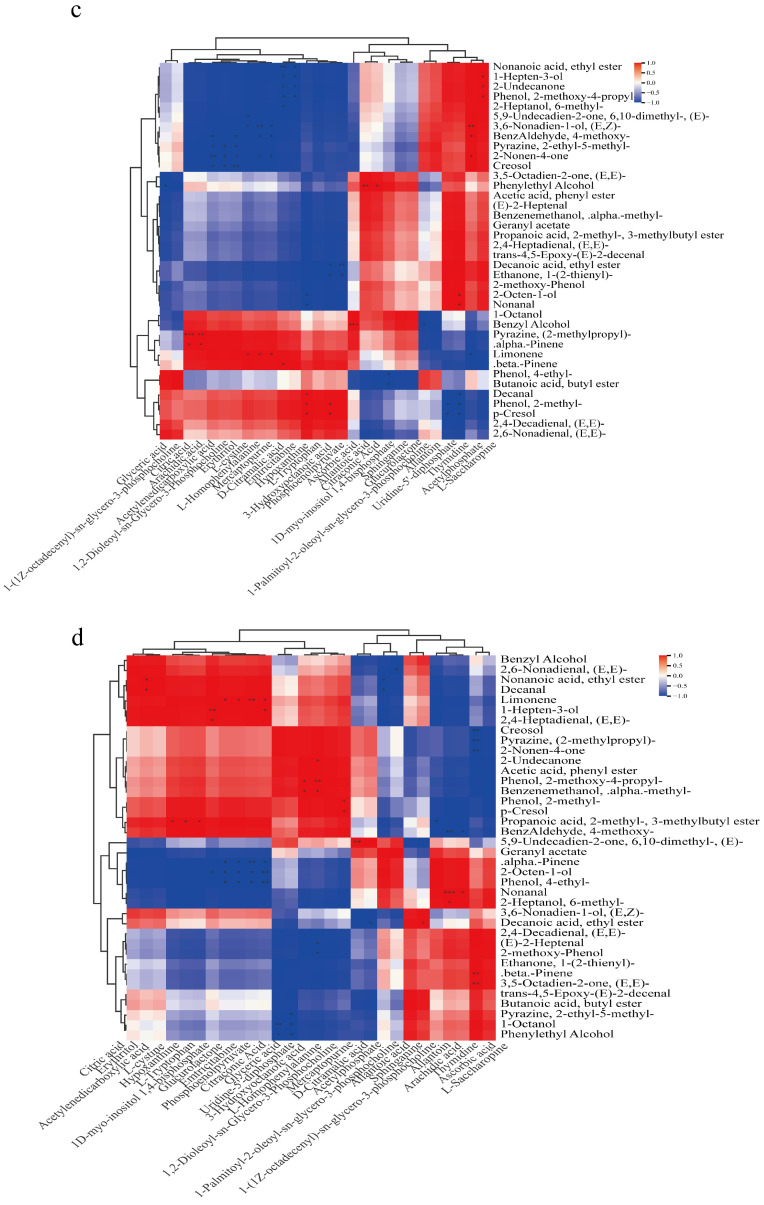
VIP values of the top 10 genera at the genus level (**A**). Redundancy analysis among microorganisms, VOCs, and differential metabolites (**B**), where red arrows represent microorganisms, blue arrows represent differential metabolites, and green represents key volatile substances. Heat map of the correlation between differential metabolites and key VOCs of sour meat in different groups (**C**), where (**a**) is group C, (**b**) is group LP, (**c**) is group K-Ca, and (**d**) is group LP + K-Ca. *, **, and *** indicate significant correlations at *p* < 0.05, *p* < 0.01, and *p* < 0.001, respectively.

In order to investigate flavor variations in sour meat more comprehensively, the ten most abundant microbial genera were selected and analyzed using the OPLS-DA model, resulting in 5 microorganisms with VIP > 1 (Figure 5A). Redundancy analysis was performed between these 5 microorganisms, 38 key volatile compounds with VIP > 1, and 28 differential metabolites with VIP > 1.5 (Figure 5B). The results revealed that the *Macrococcus, Staphylococcus* and *Lactiplantibacillus* were significantly correlated with volatile compounds and metabolites (*p* < 0.05). A clustered heatmap was generated to visually represent the correlation between VOCs and metabolites (Figure 5C).

As shown in Figure 5B,C, p-cresol showed a negative association with *L. plantarum*, while demonstrating positive correlations with *Macrococcus* and *Staphylococcus*. This suggested that the *L. plantarum* SJ-4-dominated community may help reduce the accumulation of this undesirable aromatic compound [77]. In group C, 1-palmitoyl-2-oleoyl-sn-glycero-3-phosphocholine and 1-(1Z-octadecenyl)-sn-glycero-3-phosphocholine exhibited a positive correlation with volatile flavor substances (including 4-methoxybenzaldehyde, 2-Octen-1-ol, and p-cresol). These metabolites also showed a positive correlation with *Staphylococcus* and *Macrococcus*. This may be related to the lipolytic activity of *Staphylococcus* and *Macrococcus*, which can promote the release of lipid-derived flavor precursors [78]. In the LP + K-Ca group, metabolites such as ascorbic acid, L-cystine, arachidic acid, and citraconic acid were positively correlated with several flavor compounds and *Lactiplantibacillus*. This association may reflect the role of *L. plantarum* SJ-4 in regulating amino acid, organic acid, and lipid metabolism, while CaA may contribute to maintaining oxidative stability. These correlations further supported that the combined treatment reshaped microbial metabolism, metabolic pathways and promoted the formation of a more balanced flavor profile [79,80].

However, this study has some limitations that should be acknowledged. The fermentation experiments were conducted under specific conditions, so the applicability of the results to different raw materials and processing conditions needs further verification. In addition, consumer acceptance and storage stability of the product still require further evaluation. Future studies should focus on the sensory characteristics preferred by consumers and the stability of reduced-salt sour meat during storage, so as to support its practical application. Furthermore, although ginger and Sichuan pepper were uniformly added to all treatments as common background factors, their independent effects on sour meat fermentation were not specifically investigated. Future studies should further clarify their roles in microbial succession, fermentation stability, and flavor formation by setting spice-free or single-spice control groups.

## 4. Conclusions

In this study, a comprehensive analysis was conducted on volatile flavor compounds, microbial communities, and metabolites in sour meat. The results showed that the pH value of the LP + K-Ca group was significantly lower than that of other groups, accompanied by a higher total acid content, indicating that the combined treatment of salt substitution and inoculation with *L. plantarum* SJ-4 promoted rapid acidification. LP + K-Ca exhibited the lowest nitrite level, indicating that the combined treatment inhibited nitrite accumulation. Results from E-tongue, E-nose, FAAs and VOCs analyses demonstrated that combined treatment not only improved the overall flavor profile but also effectively reduced the production of unpleasant off-flavors. Analysis of microbial community dynamics confirmed the dominant role of lactic acid bacteria in the sour meat fermentation system. Among these, *Macrococcus*, *Staphylococcus* and *Lactiplantibacillus* were identified as the key functional microorganisms driving flavor differentiation in sour meat. Further non-targeted metabolomics analysis revealed that the differential regulation of amino acid metabolism, organic acid metabolism, and lipid metabolism pathways may contribute to the variations in flavor profiles observed among the different treatment groups. Overall, this study demonstrates that the combined application of 24% KCl and 16% CaA, together with inoculation of 2% *L. plantarum* SJ-4, showed potential to shorten fermentation to 25 days, improve flavor formation, and enhance fermentation safety. This study provides a useful reference for the optimization of sour meat fermentation technology and flavor regulation and offers a promising strategy for the development of salt-reduced sour meat based on partial NaCl replacement.

## Figures and Tables

**Table 1:** Response characteristics of each sensor of the electronic nose.

Sensors	Description
W1C	Sensitive to aromatic and benzene ingredients
W5S	Sensitive to nitrogen oxides
W3C	Sensitive to aromatic and ammonia ingredients
W6S	Selective to hydrogen
W5C	Sensitive to short-chain alkane aromatic compounds
W1S	Sensitive to methyl
W1W	Sensitive to sulfides
W2S	Sensitive to alcohols, aldehydes, ethers and ketones
W2W	Sensitive to organic sulfides and aromatic ingredients
W3S	Sensitive to alkanes

**Table 2:** Changes in free amino acids (FAAs) concentration of sour meat (mg/100 g).

Items	0d	25d			
		C	LP	K-Ca	LP + K-Ca
Asp	3.15 ± 1.13 ^d^	21.26 ± 1.00 ^b^	23.77 ± 0.40 ^a^	17.84 ± 0.15 ^c^	21.10 ± 0.06 ^b^
Thr	0.68 ± 0.14 ^c^	10.48 ± 0.68 ^b^	12.00 ± 0.17 ^a^	12.36 ± 1.00 ^a^	12.99 ± 0.21 ^a^
Ser	1.00 ± 0.45 ^b^	3.06 ± 0.50 ^a^	0.95 ± 0.15 ^b^	0.80 ± 0.27 ^b^	0.95 ± 0.28 ^b^
Glu	6.10 ± 0.63 ^e^	72.86 ± 0.13 ^d^	77.67 ± 0.71 ^b^	74.47 ± 0.91 ^c^	89.78 ± 1.46 ^a^
Gly	6.03 ± 0.69 ^d^	19.22 ± 0.03 ^a^	16.90 ± 0.04 ^c^	17.49 ± 0.02 ^b^	18.72 ± 0.01 ^a^
Ala	15.45 ± 0.38 ^c^	44.52 ± 0.22 ^b^	45.27 ± 0.06 ^b^	44.75 ± 0.05 ^b^	47.09 ± 0.10 ^a^
Cys	0.51 ± 0.10 ^d^	1.12 ± 0.07 ^b^	0.82 ± 0.07 ^c^	1.25 ± 0.04 ^a^	0.75 ± 0.02 ^c^
Val	4.06 ± 0.10 ^c^	23.48 ± 0.11 ^b^	23.88 ± 0.15 ^a^	23.82 ± 0.02 ^a^	23.78 ± 0.06 ^a^
Met	3.60 ± 3.07 ^c^	11.59 ± 0.07 ^ab^	17.15 ± 4.31 ^a^	6.61 ± 0.34 ^bc^	13.33 ± 0.22 ^a^
Ile	12.70 ± 2.96 ^b^	20.49 ± 0.31 ^ab^	28.07 ± 0.37 ^a^	23.11 ± 0.11 ^ab^	33.06 ± 8.42 ^a^
Leu	3.65 ± 1.83 ^d^	35.80 ± 0.63 ^c^	47.07 ± 2.03 ^a^	41.54 ± 0.25 ^b^	49.13 ± 1.74 ^a^
Tyr	3.59 ± 0.21 ^c^	3.16 ± 0.17 ^d^	4.34 ± 0.08 ^b^	3.09 ± 0.16 ^d^	4.74 ± 0.10 ^a^
Phe	5.90 ± 2.34 ^c^	23.37 ± 0.16 ^b^	27.02 ± 0.03 ^a^	25.59 ± 0.04 ^a^	27.20 ± 0.11 ^a^
His	321.56 ± 0.38 ^a^	296.08 ± 0.93 ^b^	272.23 ± 0.25 ^d^	296.84 ± 0.22 ^b^	275.75 ± 0.77 ^c^
Lys	5.87 ± 1.11 ^d^	35.25 ± 0.42 ^b^	27.81 ± 0.17 ^c^	35.39 ± 0.13 ^b^	37.42 ± 0.12 ^a^
Arg	3.19 ± 0.17 ^a^	1.35 ± 0.15 ^d^	2.08 ± 0.26 ^c^	1.38 ± 0.05 ^d^	2.39 ± 0.14 ^b^
Pro	1.27 ± 0.02 ^c^	21.34 ± 0.50 ^a^	17.21 ± 0.52 ^b^	17.56 ± 0.37 ^b^	18.18 ± 0.20 ^b^
EAAs	36.46 ± 11.55 ^d^	160.46 ± 2.37 ^c^	183.00 ± 7.22 ^ab^	168.42 ± 1.87 ^bc^	196.90 ± 10.87 ^a^
TFAAs	398.31 ± 15.70 ^c^	644.43 ± 6.06 ^b^	644.25 ± 9.76 ^b^	643.90 ± 4.10 ^b^	676.33 ± 14.00 ^a^

All values are mean ± standard deviation of three replicates (*n* = 3). ^a–d^ Means with different lowercase letters within the same row were significantly different (*p* < 0.05). EAAs: essential amino acids; TFAAs: total free amino acids. C (control group), LP (inoculation group), K-Ca (substitution group) and LP + K-Ca (combined treatment group).

**Table 3:** Volatile flavor compounds concentration in sour meat at the end of fermentation (μg/g).

NO.	Compounds	Groups			
		C	LP	K-Ca	LP + K-Ca
V1	1-Hepten-3-ol	0.0027 ± 0.0018 ^c^	0.0155 ± 0.0042 ^b^	0.0112 ± 0.0007 ^b^	0.0216 ± 0.0002 ^a^
V2	2-Undecanol	0.0063 ± 0.0008 ^ab^	0.0073 ± 0.0026 ^ab^	0.0044 ± 0.0009 ^b^	0.0096 ± 0.0019 ^a^
V3	5-methyl-2-(1-methylethyl)-Cyclohexanol	0.5042 ± 0.0259 ^b^	0.5505 ± 0.0591 ^ab^	0.4280 ± 0.0270 ^c^	0.6052 ± 0.0246 ^a^
V4	3-Hexen-1-ol, acetate, (Z)-	0.0609 ± 0.0034 ^b^	0.0783 ± 0.0056 ^a^	0.0596 ± 0.0086 ^b^	0.0810 ± 0.0081 ^a^
V5	Benzenemethanol, .alpha.-methyl-	0.9728 ± 0.1280 ^a^	0.7401 ± 0.1496 ^b^	0.5322 ± 0.0267 ^c^	0.8699 ± 0.0537 ^ab^
V6	Hotrienol	0.2287 ± 0.0162 ^a^	0.1986 ± 0.0121 ^b^	0.1464 ± 0.0086 ^c^	0.2068 ± 0.0108 ^ab^
V7	2-Heptanol, 6-methyl-	0.0783 ± 0.0009 ^b^	0.0843 ± 0.0045 ^b^	0.0983 ± 0.0025 ^a^	0.0962 ± 0.0074 ^a^
V8	Geraniol	0.0195 ± 0.0002 ^a^	0.0192 ± 0.0047 ^a^	0.0186 ± 0.0009 ^a^	0.0193 ± 0.0007 ^a^
V9	.alpha.-Terpineol	1.1686 ± 0.0896 ^a^	1.0144 ± 0.1466 ^a^	0.7767 ± 0.0488 ^b^	0.9911 ± 0.0772 ^a^
V10	6-Undecanol	0.0327 ± 0.0019 ^b^	0.0229 ± 0.0135 ^b^	0.0202 ± 0.0020 ^b^	0.0759 ± 0.0021 ^a^
V11	dl-Menthol	0.5042 ± 0.0259 ^b^	0.5505 ± 0.0591 ^ab^	0.4280 ± 0.0270 ^c^	0.6052 ± 0.0246 ^a^
V12	Benzyl Alcohol	0.4302 ± 0.0323 ^a^	0.4340 ± 0.0104 ^a^	0.3222 ± 0.0169 ^c^	0.3726 ± 0.0047 ^b^
V13	2-Decanol	0.1137 ± 0.0076 ^a^	0.0951 ± 0.0117 ^b^	0.0778 ± 0.0041 ^c^	0.0914 ± 0.0055 ^bc^
V14	1-Octanol	0.1404 ± 0.0088 ^c^	0.1967 ± 0.0060 ^a^	0.1261 ± 0.0042 ^c^	0.1720 ± 0.0101 ^b^
V15	Terpinen-4-ol	7.9155 ± 0.3812 ^bc^	8.8695 ± 1.1086 ^b^	6.8454 ± 0.3890 ^c^	10.1378 ± 0.4743 ^a^
V16	3-Cyclohexen-1-ol, 4-methyl-1-(1-methylethyl)-, (R)-	7.9155 ± 0.3812 ^bc^	8.8695 ± 1.1086 ^b^	6.8454 ± 0.3890 ^c^	10.1378 ± 0.4743 ^a^
V17	2-Octen-1-ol	0.9607 ± 0.0768 ^a^	0.4012 ± 0.0737 ^b^	0.5057 ± 0.0133 ^b^	0.4061 ± 0.0260 ^b^
V18	Phenylethyl Alcohol	3.1117 ± 0.3490 ^ab^	2.7383 ± 0.7083 ^b^	2.4264 ± 0.0349 ^b^	3.5223 ± 0.0217 ^a^
V19	3,6-Nonadien-1-ol, (E,Z)-	0.0691 ± 0.0097 ^a^	0.0112 ± 0.0012 ^c^	0.0504 ± 0.0037 ^b^	0.0156 ± 0.0013 ^c^
V20	1-Nonanol	0.2079 ± 0.0296 ^ab^	0.1727 ± 0.0532 ^ab^	0.2122 ± 0.0459 ^a^	0.1333 ± 0.0134 ^b^
V21	3-Furanmethanol	0.5153 ± 0.0176 ^a^	0.5224 ± 0.0262 ^a^	0.4980 ± 0.0102 ^a^	0.5046 ± 0.0191 ^a^
V22	Phenol, 2-methoxy-4-propyl-	0.0031 ± 0.0004 ^b^	0.0044 ± 0.0011 ^a^	0.0013 ± 0.0002 ^c^	0.0024 ± 0.0002 ^b^
V23	Phenol, 4-ethyl-	0.0853 ± 0.0147 ^c^	0.4895 ± 0.0910 ^a^	0.3330 ± 0.0409 ^b^	0.4698 ± 0.0483 ^a^
V24	Phenol, 3,4-dimethyl-	1.8143 ± 0.1391 ^a^	1.5699 ± 0.2227 ^ab^	1.1862 ± 0.0747 ^c^	1.5209 ± 0.1164 ^b^
V25	Phenol, 2-ethyl-	0.0456 ± 0.0014 ^a^	0.0481 ± 0.0073 ^a^	0.0276 ± 0.0029 ^b^	0.0470 ± 0.0043 ^a^
V26	Creosol	1.6500 ± 0.1712 ^ab^	1.6911 ± 0.2933 ^ab^	1.3343 ± 0.0803 ^b^	1.9701 ± 0.2159 ^a^
V27	Phenol, 2,6-dimethyl-	5.6915 ± 0.6282 ^a^	5.0605 ± 1.2425 ^ab^	4.0804 ± 0.3640 ^b^	5.9818 ± 0.4116 ^a^
V28	Phenol, 2-methyl-	0.1549 ± 0.0125 ^a^	0.0713 ± 0.0111 ^b^	0.0838 ± 0.0022 ^b^	0.0788 ± 0.0022 ^b^
V29	2-methoxy-Phenol	0.1563 ± 0.0105 ^a^	0.1575 ± 0.0087 ^a^	0.1138 ± 0.0037 ^c^	0.1417 ± 0.0057 ^b^
V30	2-Methoxy-5-methylphenol	2.0118 ± 0.1442 ^a^	1.6911 ± 0.2933 ^ab^	1.3343 ± 0.0803 ^c^	1.6083 ± 0.0990 ^bc^
V31	p-Cresol	0.1549 ± 0.0125 ^a^	0.0713 ± 0.0111 ^b^	0.0838 ± 0.0022 ^b^	0.0788 ± 0.0022 ^b^
V32	(E)-2-Decenal	0.0010 ± 0.0008 ^b^	0.0036 ± 0.0030 ^b^	0.0016 ± 0.0004 ^b^	0.0209 ± 0.0004 ^a^
V33	(E)-2-Heptenal	0.0085 ± 0.0016 ^b^	0.0092 ± 0.0010 ^b^	0.0200 ± 0.0021 ^a^	0.0177 ± 0.0028 ^a^
V34	trans-4,5-Epoxy-(E)-2-decenal	0.0584 ± 0.0012 ^b^	0.0818 ± 0.0210 ^a^	0.0269 ± 0.0017 ^c^	0.0439 ± 0.0002 ^bc^
V35	2,4-Heptadienal, (E,E)-	0.1121 ± 0.0137 ^a^	0.0817 ± 0.0125 ^b^	0.0571 ± 0.0027 ^c^	0.0897 ± 0.0082 ^b^
V36	Benzaldehyde, 3-methoxy-	0.3787 ± 0.0255 ^a^	0.3422 ± 0.0481 ^a^	0.2683 ± 0.0142 ^b^	0.3371 ± 0.0201 ^a^
V37	2-Undecenal	0.0258 ± 0.0025 ^a^	0.0345 ± 0.0110 ^a^	0.0132 ± 0.0018 ^b^	0.0296 ± 0.0037 ^a^
V38	6-Octenal, 3,7-dimethyl-, (R)-	0.1025 ± 0.0102 ^a^	0.0851 ± 0.0081 ^bc^	0.0782 ± 0.0044 ^c^	0.0931 ± 0.0048 ^ab^
V39	2-Heptenal, 2-propyl-	1.2520 ± 0.0611 ^b^	1.3671 ± 0.1595 ^ab^	1.0606 ± 0.0570 ^c^	1.5407 ± 0.0653 ^a^
V40	(E)-2-Octenal	0.0264 ± 0.0017 ^b^	0.0168 ± 0.0083 ^c^	0.0195 ± 0.0004 ^bc^	0.0567 ± 0.0011 ^a^
V41	Hexanal, 2-ethyl-	1.1992 ± 0.0635 ^a^	1.2798 ± 0.0554 ^a^	1.2334 ± 0.0421 ^a^	1.2319 ± 0.1004 ^a^
V42	2,6-Nonadienal, (E,E)-	0.0336 ± 0.0013 ^a^	0.0243 ± 0.0023 ^b^	0.0272 ± 0.0031 ^b^	0.0250 ± 0.0022 ^b^
V43	2,4-Decadienal, (E,E)-	0.0068 ± 0.0002 ^a^	0.0052 ± 0.0005 ^b^	0.0035 ± 0.0005 ^c^	0.0047 ± 0.0003 ^b^
V44	Nonanal	0.1548 ± 0.0110 ^b^	0.1598 ± 0.0175 ^b^	0.2117 ± 0.0114 ^a^	0.2337 ± 0.0120 ^a^
V45	(2E,4Z)-2,4-Decadienal	0.0141 ± 0.0014 ^a^	0.0138 ± 0.0050 ^a^	0.0067 ± 0.0004 ^b^	0.0143 ± 0.0037 ^a^
V46	Decanal	0.0452 ± 0.0078 ^a^	0.0199 ± 0.0011 ^c^	0.0236 ± 0.0016 ^bc^	0.0302 ± 0.0040 ^b^
V47	2,4-Nonadienal, (E,E)-	0.0942 ± 0.0128 ^a^	0.1161 ± 0.0167 ^a^	0.0601 ± 0.0074 ^b^	0.1061 ± 0.0193 ^a^
V48	cis-4-Decenal	2.4469 ± 0.1332 ^bc^	2.7715 ± 0.3234 ^b^	2.1650 ± 0.1139 ^c^	3.2815 ± 0.1206 ^a^
V49	2-Nonenal	0.2778 ± 0.1564 ^a^	0.0604 ± 0.0070 ^b^	0.0894 ± 0.0052 ^b^	0.0738 ± 0.0008 ^b^
V50	BenzAldehyde, 4-methoxy-	0.3179 ± 0.0232 ^b^	0.4340 ± 0.0581 ^a^	0.2078 ± 0.0077 ^c^	0.2942 ± 0.0182 ^b^
V51	2,4-Nonadienal	0.1219 ± 0.0084 ^ab^	0.1614 ± 0.0527 ^a^	0.0710 ± 0.0049 ^b^	0.1502 ± 0.0109 ^a^
V52	2,6-Nonadienal, (E,Z)-	0.0336 ± 0.0013 ^a^	0.0243 ± 0.0023 ^b^	0.0272 ± 0.0031 ^b^	0.0250 ± 0.0022 ^b^
V53	Butanoic acid, butyl ester	0.0193 ± 0.0026 ^b^	0.0292 ± 0.0061 ^a^	0.0174 ± 0.0001 ^b^	0.0260 ± 0.0015 ^a^
V54	Formic acid, 2-phenylethyl ester	0.2971 ± 0.0121 ^b^	0.3274 ± 0.0388 ^ab^	0.2514 ± 0.0137 ^c^	0.3662 ± 0.0153 ^a^
V55	Propanoic acid, pentyl ester	0.0984 ± 0.0072 ^a^	0.1090 ± 0.0077 ^a^	0.0810 ± 0.0073 ^b^	0.1129 ± 0.0080 ^a^
V56	Propanoic acid, 2-methyl-, 3-methylbutyl ester	0.1258 ± 0.0160 ^a^	0.0899 ± 0.0145 ^b^	0.0608 ± 0.0026 ^c^	0.0958 ± 0.0057 ^b^
V57	Geranyl acetate	0.1540 ± 0.0054 ^b^	0.2046 ± 0.0504 ^a^	0.0669 ± 0.0051 ^c^	0.1137 ± 0.0027 ^bc^
V58	Nonanoic acid, ethyl ester	0.0216 ± 0.0034 ^a^	0.0057 ± 0.0010 ^bc^	0.0086 ± 0.0011 ^b^	0.0045 ± 0.0011 ^c^
V59	Pentanoic acid, 4-methyl-, ethyl ester	0.0127 ± 0.0018 ^a^	0.0127 ± 0.0021 ^a^	0.0094 ± 0.0048 ^a^	0.0091 ± 0.0023 ^a^
V60	2(3H)-Furanone, 5-butyldihydro-	0.0335 ± 0.0010 ^c^	0.1363 ± 0.0030 ^b^	0.0399 ± 0.0058 ^c^	0.3391 ± 0.0052 ^a^
V61	Decanoic acid, ethyl ester	0.0204 ± 0.0014 ^a^	0.0053 ± 0.0019 ^c^	0.0121 ± 0.0010 ^b^	0.0043 ± 0.0003 ^c^
V62	Heptanoic acid, methyl ester	0.0172 ± 0.0021 ^a^	0.0153 ± 0.0031 ^a^	0.0144 ± 0.0004 ^a^	0.0135 ± 0.0010 ^a^
V63	Acetic acid, phenyl ester	1.0945 ± 0.1459 ^a^	0.8400 ± 0.1682 ^b^	0.6043 ± 0.0316 ^c^	0.9895 ± 0.0544 ^ab^
V64	n-Butyl methacrylate	0.2171 ± 0.0136 ^a^	0.0883 ± 0.1168 ^bc^	0.1442 ± 0.0086 ^ab^	0.0196 ± 0.0006 ^c^
V65	Methyl anthranilate	0.1109 ± 0.0048 ^a^	0.0782 ± 0.0313 ^ab^	0.0469 ± 0.0016 ^b^	0.0832 ± 0.0106 ^a^
V66	3-Mercaptohexyl acetate	0.0027 ± 0.0002 ^a^	0.0039 ± 0.0014 ^a^	0.0030 ± 0.0008 ^a^	0.0026 ± 0.0003 ^a^
V67	Benzoic acid, methyl ester	0.8000 ± 0.0623 ^a^	0.7497 ± 0.0457 ^ab^	0.5254 ± 0.0179 ^c^	0.6996 ± 0.0337 ^b^
V68	5,9-Undecadien-2-one, 6,10-dimethyl-, (E)-	0.0049 ± 0.0006 ^b^	0.0040 ± 0.0031 ^b^	0.0108 ± 0.0029 ^a^	0.0108 ± 0.0004 ^a^
V69	3-Dodecanone	0.0163 ± 0.0101 ^a^	0.0079 ± 0.0034 ^a^	0.0166 ± 0.0003 ^a^	0.0061 ± 0.0003 ^a^
V70	2-Nonen-4-one	0.0559 ± 0.0033 ^a^	0.0580 ± 0.0070 ^a^	0.0415 ± 0.0045 ^b^	0.064 ± 0.0067 ^a^
V71	2-Butanone, 4-(2,6,6-trimethyl-2-cyclohexen-1-yl)-	0.0045 ± 0.0013 ^a^	0.0026 ± 0.0009 ^ab^	0.0012 ± 0.0001 ^b^	0.0027 ± 0.0011 ^ab^
V72	(-)-Carvone	0.3285 ± 0.0236 ^b^	0.4187 ± 0.0476 ^a^	0.2916 ± 0.0184 ^b^	0.4488 ± 0.0588 ^a^
V73	Carvone	0.3093 ± 0.0022 ^a^	0.3408 ± 0.0789 ^a^	0.3125 ± 0.0111 ^a^	0.3232 ± 0.0049 ^a^
V74	5-Hepten-2-one, 6-methyl-	0.3344 ± 0.0529 ^a^	0.2869 ± 0.0645 ^ab^	0.1204 ± 0.0068 ^c^	0.2330 ± 0.0052 ^b^
V75	2-Undecanone	0.0308 ± 0.0042 ^a^	0.0117 ± 0.0025 ^c^	0.0218 ± 0.0028 ^b^	0.0089 ± 0.0007 ^c^
V76	α-Ionone, 6-methyl-	0.0120 ± 0.0019 ^a^	0.0098 ± 0.0034 ^a^	0.0051 ± 0.0008 ^b^	0.0122 ± 0.0037 ^a^
V77	2-Cyclohexen-1-one, 3-methyl-6-(1-methylethyl)-	8.6538 ± 0.6144 ^a^	9.4981 ± 0.9175 ^a^	6.7796 ± 0.3231 ^b^	9.3402 ± 0.9345 ^a^
V78	1-(4-methylphenyl)-Ethanone	0.2679 ± 0.0135 ^b^	0.2892 ± 0.0289 ^ab^	0.2351 ± 0.0109 ^c^	0.3179 ± 0.0080 ^a^
V79	5-Nonanone	0.1115 ± 0.0081 ^b^	0.1446 ± 0.0212 ^a^	0.1137 ± 0.0007 ^b^	0.1435 ± 0.0066 ^a^
V80	2-Cyclopenten-1-one, 2-hydroxy-3-methyl-	8.7804 ± 0.8289 ^a^	8.2931 ± 0.5529 ^a^	6.4468 ± 0.2765 ^b^	7.2382 ± 0.0643 ^b^
V81	D-Carvone	0.3285 ± 0.0236 ^b^	0.4187 ± 0.0476 ^a^	0.2916 ± 0.0184 ^b^	0.4488 ± 0.0588 ^a^
V82	L-Fenchone	3.8666 ± 0.2921 ^a^	3.7771 ± 0.1789 ^ab^	2.6435 ± 0.0837 ^c^	3.4796 ± 0.1492 ^b^
V83	Ethanone, 1-(4,5-dihydro-2-thiazolyl)-	0.0554 ± 0.0024 ^a^	0.0519 ± 0.0061 ^ab^	0.0464 ± 0.0008 ^b^	0.0496 ± 0.0026 ^ab^
V84	Ethanone, 1-(2-aminophenyl)-	0.0190 ± 0.0016 ^a^	0.0127 ± 0.0044 ^bc^	0.0091 ± 0.0006 ^c^	0.0149 ± 0.0017 ^ab^
V85	3,5-Octadien-2-one, (E,E)-	0.0474 ± 0.0038 ^b^	0.0574 ± 0.0052 ^a^	0.0404 ± 0.0007 ^b^	0.0455 ± 0.0043 ^b^
V86	Ethanone, 1-(2-thienyl)-	0.1213 ± 0.0052 ^bc^	0.1469 ± 0.0085 ^a^	0.1115 ± 0.0033 ^c^	0.1267 ± 0.0105 ^b^
V87	2H-Pyran-2-one, tetrahydro-6-methyl-	4.3691 ± 0.2947 ^a^	4.2652 ± 0.2331 ^a^	3.1877 ± 0.0609 ^b^	4.1080 ± 0.0504 ^a^
V88	2(5H)-Furanone, 3-hydroxy-4,5-dimethyl-	2.9118 ± 0.1701 ^a^	2.8142 ± 0.2144 ^a^	2.2580 ± 0.0391 ^b^	2.7016 ± 0.0877 ^a^
V89	1,2-Cyclopentanedione, 3-methyl-	13.1094 ± 1.3382 ^a^	12.4024 ± 1.0840 ^a^	9.6738 ± 0.4536 ^b^	10.6540 ± 0.1098 ^b^
V90	1-Octen-3-one	0.0046 ± 0.0008 ^a^	0.0048 ± 0.0005 ^a^	0.0045 ± 0.0005 ^ab^	0.0035 ± 0.0003 ^b^
V91	Cyclohexanone, 2,2,6-trimethyl-	2.7262 ± 0.2676 ^a^	2.5523 ± 0.1654 ^a^	1.9517 ± 0.0888 ^b^	2.2089 ± 0.0148 ^b^
V92	Pyrazine, (2-methylpropyl)-	0.5278 ± 0.0057 ^b^	0.7046 ± 0.0898 ^a^	0.2989 ± 0.0168 ^c^	0.7914 ± 0.0698 ^a^
V93	Pyrazine, 2-ethyl-5-methyl-	0.0844 ± 0.0086 ^a^	0.0443 ± 0.0019 ^b^	0.0873 ± 0.0054 ^a^	0.0375 ± 0.0068 ^b^
V94	Furan, 2-pentyl-	0.2843 ± 0.0067 ^b^	0.3751 ± 0.0911 ^ab^	0.1433 ± 0.0096 ^c^	0.4249 ± 0.0547 ^a^
V95	2-Acetylthiazole	9.9826 ± 0.0972 ^b^	11.5775 ± 0.9914 ^a^	9.0865 ± 0.4111 ^b^	12.1809 ± 1.2186 ^a^
V96	Pyridine, 2-pentyl-	8.1670 ± 0.6791 ^a^	8.4199 ± 1.2347 ^a^	6.3890 ± 0.4092 ^b^	9.7683 ± 0.7520 ^a^
V97	Pyrazine, 2-methoxy-3-(2-methylpropyl)-	6.5417 ± 0.1785 ^a^	6.3425 ± 1.3749 ^a^	6.1250 ± 0.4318 ^a^	6.3872 ± 0.0869 ^a^
V98	Benzyl methyl sulfide	3.1862 ± 0.1679 ^b^	3.4562 ± 0.3849 ^ab^	2.7244 ± 0.1298 ^c^	3.8826 ± 0.1480 ^a^
V99	DiSulfur compounds, dipropyl	0.4384 ± 0.0266 ^a^	0.4232 ± 0.0280 ^a^	0.3219 ± 0.0065 ^b^	0.3992 ± 0.01280 ^a^
V100	β-Pinene	0.5040 ± 0.0675 ^a^	0.3384 ± 0.0539 ^b^	0.2202 ± 0.0071 ^c^	0.3189 ± 0.0106 ^b^
V101	α-Pinene	1.0909 ± 0.1543 ^a^	0.6514 ± 0.1256 ^b^	0.3332 ± 0.0203 ^c^	0.5441 ± 0.0671 ^b^
V102	.alpha.-Phellandrene 1	0.4589 ± 0.0322 ^a^	0.4254 ± 0.0354 ^a^	0.2918 ± 0.0043 ^c^	0.3757 ± 0.0189 ^b^
V103	.beta.-Phellandrene	5.4424 ± 0.4718 ^a^	4.6491 ± 0.4960 ^b^	2.7475 ± 0.0799 ^c^	4.4475 ± 0.0020 ^b^
V104	D-Limonene	5.2001 ± 0.4698 ^a^	4.5609 ± 0.2710 ^b^	2.7672 ± 0.1168 ^c^	4.2126 ± 0.0235 ^b^
V105	Limonene	5.2001 ± 0.4698 ^a^	4.5609 ± 0.2710 ^b^	2.7672 ± 0.1168 ^d^	3.9727 ± 0.2079 ^c^

All values are mean ± standard deviation of three replicates (*n* = 3). ^a–d^ Means with different lowercase letters within the same row were significantly different (*p* < 0.05).

**Table 4:** Metabolic pathway with significant enrichment of differential metabolites in sour meat.

Metabolite Pathways	Total	Hits	Raw *p*	−Log(*p*)	Impact	FDR
Ascorbate and aldarate metabolism	10	3	0.00400	2.39810	0.40541	0.15995
Tryptophan metabolism	41	5	0.01210	1.91720	0.33946	0.26804
Glycerolipid metabolism	16	2	0.10414	0.98238	0.33022	0.55542
Alanine, aspartate and glutamate metabolism	28	3	0.07030	1.15310	0.24520	0.55346
Retinol metabolism	17	1	0.45339	0.34353	0.21649	0.96940
Sulfur metabolism	8	1	0.24678	0.60768	0.21277	0.73121
Pentose and glucuronate interconversions	19	3	0.02587	1.58710	0.20482	0.39607
Biotin metabolism	10	1	0.29847	0.52511	0.20000	0.78985
Glycerophospholipid metabolism	36	6	0.00115	2.93770	0.18088	0.09233
Lysine degradation	30	3	0.08302	1.08080	0.15337	0.55346

## Data Availability

The original contributions presented in the study are included in the article; further inquiries can be directed to the corresponding author.

## Abbreviations

The following abbreviations are used in this manuscript:

NaCl	Sodium chloride
KCl	Potassium chloride
CaA	Calcium ascorbate
*L. plantarum*	*Lactobacillus plantarum*
FAAs	Free amino acids
VOCs	Volatile organic compounds
SFAs	Saturated fatty acids
UFAs	Unsaturated fatty acids
TCA cycle	Tricarboxylic acid cycle
E-tongue	Electronic tongue
E-nose	Electronic nose
